# Topics of Public Interest

**Published:** 1905-01

**Authors:** 


### TOPICS OF PUBLIC INTEREST.

### The Optometry Campaign is Opened Again.—Opticians Early Take the War Path.—Stirring Address of Chairman Van Fleet.

*To the Medical Profession of the State of New York:*

During the legislative session of 1904, a society of opticians, known as The Optical Society of the State of New York, petitioned the legislature to enact a law, creating a state board of examiners in optometry. Before this board would appear all persons who desired to practise optometry, which practice was defined in the bill to be enacted as "the employment of any means other than the use of drugs for the measurement of the powers of vision and the adaptation of lenses for the aid thereof." The Medical Society of the State of New York opposed this bill, and with the aid of other organisations, especially the county medical societies and the Optical League (an organisation of opticians, doing a legitimate business), secured its defeat.

Since the last election, this optical society has been forwarding to physicians in all parts of the state as well as to the members-elect of the next legislature, a document, giving reasons why a law of this kind should be enacted, and asking their endorsement.

At the time of the hearing on the optometry bill before the legislative committees of last year, the opticians presented a long list of names of physicians who had endorsed their efforts. The undersigned communicated with every name on that list, and learned that where reputable physicians had endorsed the measure it was through a misapprehension of the real purpose of the bill; and when its true character was pointed out to them, they not only withdrew their endorsements but in many cases wrote vigorous letters in opposition to it. Many of the names were fictitious, the communications sent to the addresses given being returned, marked "not found." A large number were the names of irregular practitioners, such as osteopaths, spiritualists, and the like. There is no doubt that the object of the opticians in presenting the present arguments is to obtain the endorsements of physicians, so that at the next legislative session these signatures can be used to offset the opposition which will be presented by the regularly organised medical bodies of the state. I, therefore, address the profession of the state, urging its members not only to refuse to endorse this and similar measures, but to make an effort to present to their representatives, both in the assembly and the senate, the true merits of the case, and urge their opposition to it.

The arguments presented by the opticians are very misleading. Their claim, of course, is that they desire to protect the community from incompetent people, but the fact is (as every well-informed physician must know) that they are all incompetent. They seek to create a separate profession. This they deny, but in their remarks before the legislative committees they continually used the expression "our profession." They seek the legal right, not only to apply lenses for the correction of defective vision which may or may not be due to errors of refraction, but they also seek to treat headaches, dizziness and the various other reflex phenomena which may be due to affections of the eye itself, or to affections of organs remote from the eye. They pose as being competent to make a differential diagnosis. To prepare physicians to do this work the law requires certain preliminary qualifications, a four years' course in a medical college, and a medical examination conducted by the state. Physicians themselves find that even after this preparation it is often difficult to be sure of their ground; and, if this is so, there seems to be no good reasons why opticians should be allowed to undertake the same work with any less preparation.

It seems unnecessary at this time to go into an extended argument in opposition to this bill. The effort to secure its enactment is not an honest one. Opticians know that they are violating the law in following the occupation which they are now engaged in, and they say that if their bill is enacted it will not give them any more powers than they now possess, while the fact is the enactment of the bill will give them the legal right to do what they are now doing in violation of the law. They really desire to use the legislature as a tool to put them beyond the grasp of the law; and if this is once clearly brought to the attention of our assemblymen and senators, there is no doubt what the outcome will be. We have met this and similar efforts more than once in the past, and there is no doubt that we shall meet many more such in years to come; but, from our experience, we feel justified in making the assertion that if the medical profession will present a united opposition to measures of this kind, they never will be enacted into laws in the state of New York.

FRANK VAN FLEET, M.D.,
Chairman of the Committee on Legislation of the
Medical Society of the State of New York.

NEW YORK, November 23, 1904.

60 E. Seventy-seventh St.

---

### Death From Christian Science.

[From *Medical Age*, December 10, 1904.]

A CIRCULAR recently issued by the Michigan State Board of Health says that an item has been going the rounds of the press relative to the death of William Taylor, a child, at Port Huron, alleging that "the parents believe that his death was caused by vaccination." An official report, however, to the secretary of the state board of health clearly proves that the alleged belief had no foundation in fact. The report says that a short time after vaccination the child was taken sick with bowel trouble and had no medical attendance, the parents being christian scientists. After the death of the child the coroner called in a reputable physician, who found that the vaccinated arm, aside from a small scar, was exactly the same as the other, and showed no signs of having been inflamed. The physician and coroner came to the conclusion that the child had died of enterocolitis. It appears that the parents belong to a sect whose members do not believe in vaccination nor in employing a physician, and undoubtedly would have been pleased to have had the death recorded as due to vaccination, especially as there is a suspicion as to the effect of the lack of proper medical attendance for the relief of the inflammation of the bowels.

